# Hybrid feature selection-based machine learning Classification system for the prediction of injury severity in single and multiple-vehicle accidents

**DOI:** 10.1371/journal.pone.0262941

**Published:** 2022-02-02

**Authors:** Shuguang Zhang, Afaq Khattak, Caroline Mongina Matara, Arshad Hussain, Asim Farooq

**Affiliations:** 1 CCCC Southwest Investment & Development Company Limited, Beijing, China; 2 The Key Laboratory of Road and Traffic Engineering, Ministry of Education, Tongji University, Jiading, Shanghai, China; 3 Department of Civil and Construction Engineering, University of Nairobi, Nairobi, Kenya; 4 NUST Institute of Civil Engineering, National University of Sciences and Technology, Islamabad, Pakistan; 5 Head of Department at Centre of Excellence in Transportation Engineering, Pak Austria Facshhoule, Institute of Applied Sciences, Haripur, Pakistan; Huazhong University of Science and Technology, CHINA

## Abstract

To undertake a reliable analysis of injury severity in road traffic accidents, a complete understanding of important attributes is essential. As a result of the shift from traditional statistical parametric procedures to computer-aided methods, machine learning approaches have become an important aspect in predicting the severity of road traffic injuries. The paper presents a hybrid feature selection-based machine learning classification approach for detecting significant attributes and predicting injury severity in single and multiple-vehicle accidents. To begin, we employed a Random Forests (RF) classifier in conjunction with an intrinsic wrapper-based feature selection approach called the Boruta Algorithm (BA) to find the relevant important attributes that determine injury severity. The influential attributes were then fed into a set of four classifiers to accurately predict injury severity (Naive Bayes (NB), K-Nearest Neighbor (K-NN), Binary Logistic Regression (BLR), and Extreme Gradient Boosting (XGBoost)). According to BA’s experimental investigation, the vehicle type was the most influential factor, followed by the month of the year, the driver’s age, and the alignment of the road segment. The driver’s gender, the presence of a median, and the presence of a shoulder were all found to be unimportant. According to classifier performance measures, XGBoost surpasses the other classifiers in terms of prediction performance. Using the specified attributes, the accuracy, Cohen’s Kappa, F1-Measure, and AUC-ROC values of the XGBoost were 82.10%, 0.607, 0.776, and 0.880 for single vehicle accidents and 79.52%, 0.569, 0.752, and 0.86 for multiple-vehicle accidents, respectively.

## 1. Introduction

In developing countries, road transport is the main mode of transportation for both freight and passenger traffic. In Pakistan, the railway’s influence has waned over a decade ago. Air travel due to high fares is inaccessible to the people, and the capability of water for inland transportation has not been realized at a larger scale. Over Increased reliance on the roadway network has put undue strain on the country’s highways, frequently resulting in fatalities, a situation exacerbated by their deteriorating condition. Almost no day passes without a road traffic accident on one of the country’s national highways or motorways, resulting in an increasing number of injuries and fatalities, as well as significant economic losses. Transportation safety entails avoiding collisions and minimizing the damage caused by them. Pakistan has become more mobile in recent years as a result of the construction and extension of highways. There are 2429 kilometers of highways in operation as of February 2021, with another 1312 kilometers under construction or planned. Simultaneously, the possibility of fatal accidents on our national highways and motorways becomes a greater concern. Additionally, road traffic accidents are Pakistan’s 11^th^ leading cause of premature death [1]. According to the World Health Organization’s (WHO) report, the country has 25,781 road traffic fatalities per year [2]. With the county’s growing vehicle registration, serious road safety concerns have arisen. The proportion and number of traffic fatalities have increased. Road traffic accidents have a wide range of consequences, ranging from the psychological impact on the individuals involved to the economic impact on the nation’s transportation infrastructure.

According to the World Health Organization, approximately 1.35 million people die in traffic accidents each year and this trend will have tripled by 2030 [2]. These shocking statistics demonstrate the tragic state of road accident fatalities. To improve traffic safety, a thorough investigation of the severity of traffic accidents is necessary. As a result, accurate analysis of injury severity will aid in the process of making safety decisions. Various statistical approaches have been used in the past, including multinomial logistic models, ordered probit and logit models, and mixed logit models, to predict road traffic accidents as a function of roadway, roadside, operational, and environmental factors, among other variables. Geedipally et al. [3] used a multinomial logit model to investigate motorcycle accidents. According to the researchers, factors such as alcohol consumption, lighting conditions, gender, and segment alignment (i.e., horizontal and vertical curves) all played a significant role in the accidents that occurred. Chen and Fan [4] conducted research in North Carolina and developed a multinomial logit model for estimating the severity of pedestrian-vehicle collisions. Following the results of the multinomial logit model, it was discovered that the following variables significantly increase the likelihood of fatalities and injuries: inexperienced drivers, trucks, and motorcycles; pedestrians aged 25 to 26 years old; weekends; lighting conditions (dark, dusk, and dawn); curves on the roadway; wet road surface; NC class of roadway; and speed limits between 35 and 50 miles per hour. In a study conducted using multinomial logistic regression, Vajari et al. [5] discovered that weekend crashes, motorcyclists older than 59 years, early morning/midnight crashes, multiple-vehicles involved in accidents, roundabouts, T-intersections, stop or give-way intersections, and uncontrolled intersections were all associated with a significantly increased risk of fatal accidents.

Several researchers have used ordered probit modeling to predict injury severity. Khattak et al. [6] used an ordered probit model to predict the severity of older driver injuries and found that driver gender, age, alcohol consumption, and vehicle type all play a role in crash severity in both single-and two-vehicle crashes. Kockelman and Kweon [7] investigated the risk of various injury levels in single-vehicle crashes and found that sports utility vehicles and pickup trucks are less safe than passenger cars. Abdel-Aty [8] developed an ordered probit model to predict the severity of injuries on Central Florida highway segments, toll plazas, and signalized intersections. According to the models’ findings, the gender, age, point of impact, seat belt use, vehicle type, and speed of the driver all play a role in the severity of the injury. When ordered probit modeling was used to analyze crash data from 1992 to 2001 in Singapore [9], time of day, vehicle type, road type, location type, type of collision, pedestrian age, and collision type, location type, and pedestrian age all had a significant effect on injury severity. In the analysis of driver injury severity, the Bayesian ordered probit model produced more rational parameter estimations and improved prediction performance when compared to the ordered probit model [10]. According to the ordered probit model [11], which was used to examine the effects of various factors on the severity of injury sustained by motor vehicle drivers in traffic accidents, light-vehicle drivers on two-way roads with dry road surfaces are more likely to sustain serious injury than heavy-vehicle drivers on one-way roads with wet road surfaces. The ordered probit modeling approach was used to investigate the factors that influence injury severity in downgrade crashes in Wyoming, the United States of America, which has mountainous terrain and difficult geometry [12]. The severity of the crash was affected by alcohol use, gender, vehicle maneuver, road conditions, AADT per lane, point of impact, safety equipment use, driver behavior, and car type. Multinomial logistic regression can produce imprecise predictions due to its restrictive assumption of independence of irrelevant alternatives (IIA), and it also accounts for possible correlation across repeated choices. To account for individual heterogeneity and overcome the limitations of the multinomial logit model, mixed logit models, also known as random parameter logit models, were used. Wu et al. [13] estimated the injury severity in single- and multiple-vehicle accidents on two-lane rural highways using a mixed logit model. There was a significant difference in the factors linked to injury severity in single-vehicle and multiple-vehicle crashes. There were more severe injuries and fatalities when trucks and motorcycles were involved in multi-vehicle collisions. Dim lighting and dusty weather conditions exacerbated the multiple vehicle collisions. Single-vehicle accidents were more likely when vans were used as a mode of transportation and when drivers overtook. Alcohol consumption and impaired driving were factors in both single-vehicle and multi-vehicle accidents. Chen et al. [14] used unbalanced panel data and mixed logit models to investigate the hourly likelihood of highway segments being involved in a crash. As random parameters, the traffic speed, volume, curvature, and chemically wet road surface were more accurately modelled. Low speed limits, weekends, November, and the rutting’s long remaining service life all contributed to a higher collision risk. Using a mixed logit model, Liu and Fan [15] investigated the various factors that influence the severity of head-on collisions. According to the study, young drivers’ experiences with bad weather, pickup trucks, and rural roads could be better modelled as random parameters. According to the findings, alcohol or drug use, horizontal and vertical curves, a high speed limit, motorcycle use, and elderly drivers all increased the risk of severe injury in head-on collisions. Chen et al. [16] investigated the severity of drivers’ injuries in rear-end passenger car accidents using a random parameters bivariate ordered probit modeling approach. The proposed random parameter model is outperformed by two separate ordered probit models with fixed parameters. Two drivers’ injuries, their age and gender, whether or not they used an airbag or a seat belt, and traffic flow all had a significant correlation. Deep learning and machine learning models have recently piqued academic interest in predicting the severity of injuries sustained in motor vehicle accidents. Due to their high predictive performance, machine learning-based techniques have gained a positive reputation in recent years. Zhang et al. [17] used machine learning-based algorithms to predict injury severity. They used support vector machine (SVM), decision tree (DT), K-nearest neighbor (K-NN) and random forest (RF) algorithms in addition to multinomial logit and ordered probit models. Statistical models were found to have lower predictive accuracy than machine learning classifiers.

Fiorentini and Losa [18] used DT, K-NN, RF, and LR classifiers to predict injury severity on an imbalanced dataset and a balanced dataset based on random undersampling of the majority class (RUMC). The RUMC-based models improved classifier predictability for fatal and non-fatal injuries, according to the findings. Wahab and Jiang [19] used the Classification and Regression Tree (CART) model, rule induction (PART), and Artificial Neural Network-Multilayer Perceptron to predict the severity of motorcycle accident injuries (ANN-MLP). They discovered that CART outperformed PART and ANN-MLP models in terms of overall accuracy. The study also discovered that the type of location, the time of the accident, the type of collision, and the type of settlement were the most predictive factors of injury severity. Rahim and Hassan [20] proposed a novel deep learning approach for predicting injury severity using a customized f1-loss function. Deep learning, according to the study, improved prediction performance for both fatal and non-fatal injuries. Lin et al. [21] employed four machine learning classifiers to forecast the injury severity caused by juvenile driving incidents on West Texas’ rural roadways. The speed limit, road class, and the first detrimental occurrence were the three most influential elements impacting injury severity, according to the experimental data. In addition, teen drivers’ injuries were compounded by uncontrolled and excessive speed when merging from rural roads to highways or approaching intersections, as well as refusal to yield on undivided roads with four or more lanes. To predict injury severity, Ahmadi et al. [22] used Multinomial Logistic Regression (MLR), Support Vector Machine (SVM), and Mixed-Multinomial Logit Model (MMLM), and discovered that the SVM model outperformed the others in terms of prediction accuracy. It was suggested that driver safety education, as well as vehicle and roadway design, be improved to reduce the severity of injuries.

Furthermore, traffic crash data frequently reveals an asymmetric outcome distribution, with property damage only (PDO) crashes accounting for more than 80% of all crashes and fatalities accounting for less than 1%, a problem that has gone unaddressed. Ensemble methods are superior at resolving classification problems involving unbalanced data in a variety of domains [23, 24]. Ensemble learning models combine the outputs of multiple statistical and machine learning models to obtain a better prediction estimate and range. Ji and Levinson [25] used ensemble machine learning models to predict injury severity. The results showed that the stacking model with a linear blender is preferable for the designed ensemble combination. Ensemble models outperform single models because the majority of bagging, boosting, and stacking algorithms work well. Jiang et al., [26] introduced and compared two ensemble models for modeling crash severity: AdaBoost and Gradient Boosting. Both ensemble methods outperform the MMLM and ANN models in terms of balanced prediction performance. When dealing with multiple independent attributes, a multicollinearity effect can occur because a large number of selected attributes may have the same prediction variance. To overcome this limitation, a number of researchers have reported the utility of feature selection algorithms such as principal component analysis (PCA)-based ANNs and PCA-based MLR in modeling the retention times of a variety of volatile organic compounds [27], ground-level ozone and the factors that influence its concentrations [28], and internal glasshouse humidity in North China during the winter [29], evaluation of effect of E-beam irradiation on ready-to-eat food [30], rain water quality modeling [31] and development of pistachio sorting system [32]. In the traffic and transportation domains, feature selection algorithms have been used to analyze mode choice [33], identify hotspots on roads [34], and investigate key factors affecting injury severity on rural and urban highway segments [35].

In this research, in order to obtain the important attributes as well as to deal with binary classification problem, we employed wrapper-based Boruta Algorithm as a feature selection algorithm. Following that, the machine learning classifiers including XGboost, K-NN, BLR, and NB were employed to predict the injury severity on Pakistan’s National Highway N-5. The remainder of this study is organized in the following manner. The second section discusses the overall research framework, details of statistical and machine learning classifiers and discussion on the evaluation metrics that are used in this study. Section 3 discusses the experimental analysis, which includes feature engineering via BA and performance evaluation. Conclusions and recommendations are discussed in Section 4.

## 2. Methodology

**Fig 1:**
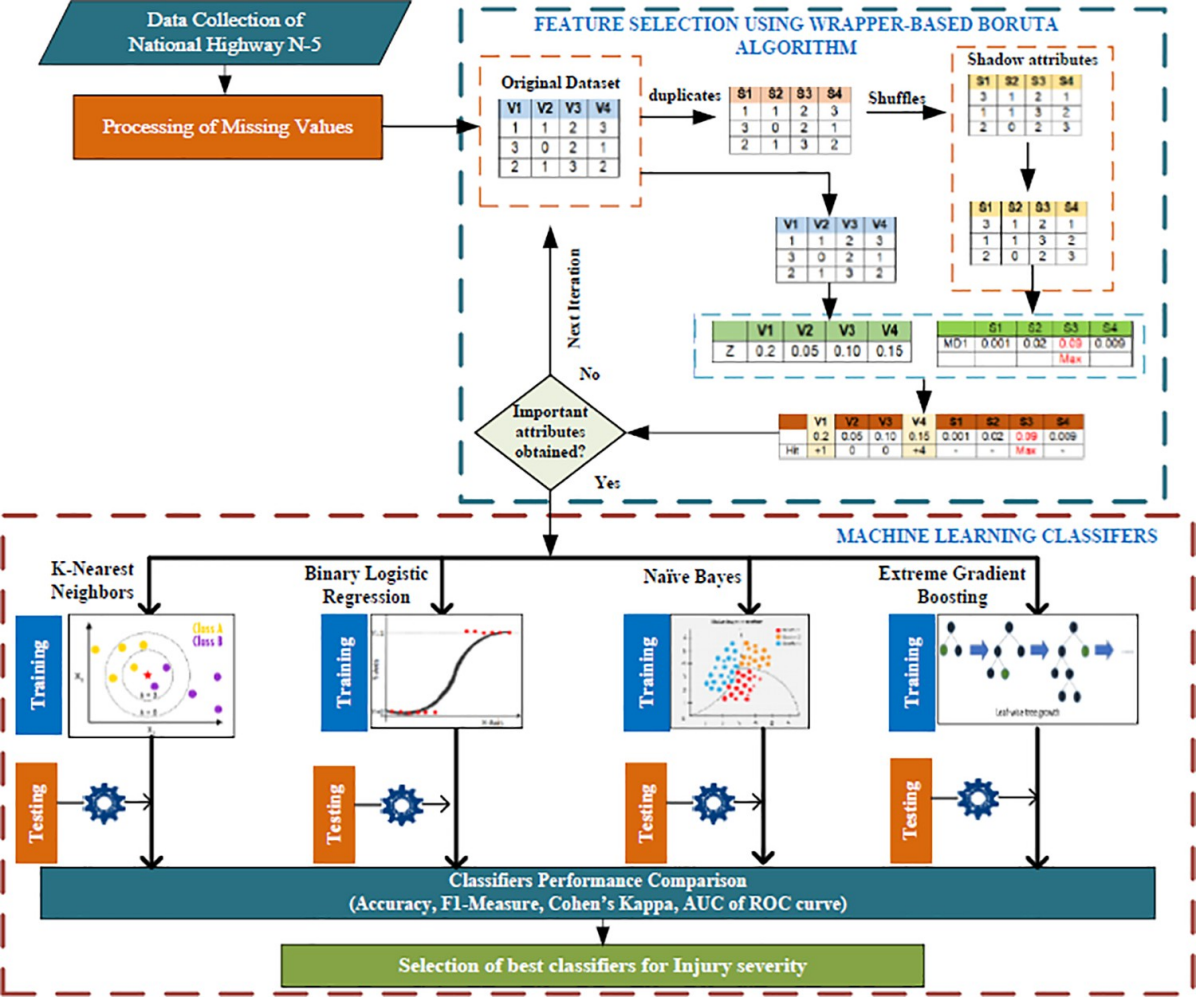
Operational framework of proposed Boruta algorithm and machine learning classification system for injury severity.

Fig 1 depicts the entire operational framework of the proposed study. There are three stages to the research. The first phase entails gathering data and preprocessing the original crash dataset, which includes removing outliers, superfluous cases, and dealing with missing data. After that, the dataset is randomly partitioned into training (80%) and testing data sets (20%). The second phase entails using the Boruta Algorithm to determine significant features (BA). The BA is a feature selection algorithm based on a wrapper that is built around the random forest (RF) classifier. It tries to capture all of the important features in a dataset that are related to a specific outcome variable. The following section contains a more in-depth discussion. After obtaining significant features via BA, the features are used as inputs to statistical and machine learning models (NB, BLR, K-NN, and XGboost) for performance evaluation in the third phase. These models are trained and then put to the test in order to predict the injury severity.

### 2.1 Study route

The purpose of this study is to determine the various factors that contribute to the injury severity of traffic accident injuries on National Highway–5 (N-5). The N-5 is a two-lane divided highway that connects Torkham in Pakistan’s Khyber Pakhtunkhwa province to Karachi in Sindh province, connecting major cities along its alignment. It is one of Pakistan’s longest highways, measuring 1819 kilometers (1310 miles) in length and passing through three provinces: Sindh, Punjab, and Khyber Pakhtunkhwa. It carries the majority of the country’s traffic, and the majority of Heavy Transport Vehicles (HTVs), use this route to transport freight from Karachi’s seaport to upcountry cities. The maximum permitted speed limit for Light Transport Vehicles (LTV), which includes passenger cars, pickup trucks, and vans, is 100 kilometers per hour. The maximum speed permitted for HTVs, which includes buses, trucks, and trailers, is 90 kilometers per hour.

### 2.2 Crash data

The data for this study came from road traffic accidents that occurred on National Highway N-5 (Peshawar-Rahim Yar Khan section) between 2015 and 2019. The data was collected from the National Highway and Motorway Police (NH&MP), which is responsible for maintaining records of road traffic accidents on Motorways and National Highways in Pakistan. The dataset included information about the injury severity, the type of collision, the cause of accidents, the time of the accident, and the vehicle type, among other things. Annexure-A contains a list of the attributes that were used in this study, as well as a description of each attribute along with its frequency and marginal percentages. Unlike motorways in Pakistan, N-5 is not an access-controlled highway and at-grade intersections are provided at various locations. For this study, we classified property damage only (PD), minor injury and major injury as non-fatal injuries, and remaining accidents that resulted in death as fatal injuries. Additionally, we have classified accident involving a single vehicle as single-vehicle accidents and those involving multiple vehicles as multiple-vehicle accidents. As a result, the problem is classified as a binary classification problem. The R programming language was used to implement the hybrid feature selection-based machine learning classification system. The number of fatal and non-fatal injuries in single- and multiple-vehicle accidents on National Highway N-5 is shown in Table 1.

**Table 1:** Number of injuries in single and multiple-vehicle accidents.

Crash type	Injury severity	No. of Injuries
Single-vehicle accident	Fatal	395
	Non-Fatal	535
Multiple-vehicle accident	Fatal	366
	Non-Fatal	488

### 2.3 Data pre-processing

Missing values for the attributes can be handled in a variety of ways, including by replacing them with the general average, by replacing them with similar type of averages, or by developing a model to predict missing values. However, in our study, we used multiple categorical attributes and the K-NN algorithm to replace missing values with neighboring values. The premise behind using K-NN to fill in missing values in our dataset is that a point’s value can be estimated using the values of the points closest to it based on other attributes.

### 2.4 Wrapper-based Boruta Algorithm (BA)

In machine learning, creating an accurate yet simple model can be difficult at times. An increase in model complexity can be caused by over-fitting or multicollinearity caused by an excessive number of attributes. A critical step in modeling and classification is the selection of attributes or features. It helps create models that are free of unwanted noise, correlated attributes, and biases.

**Fig 2:**
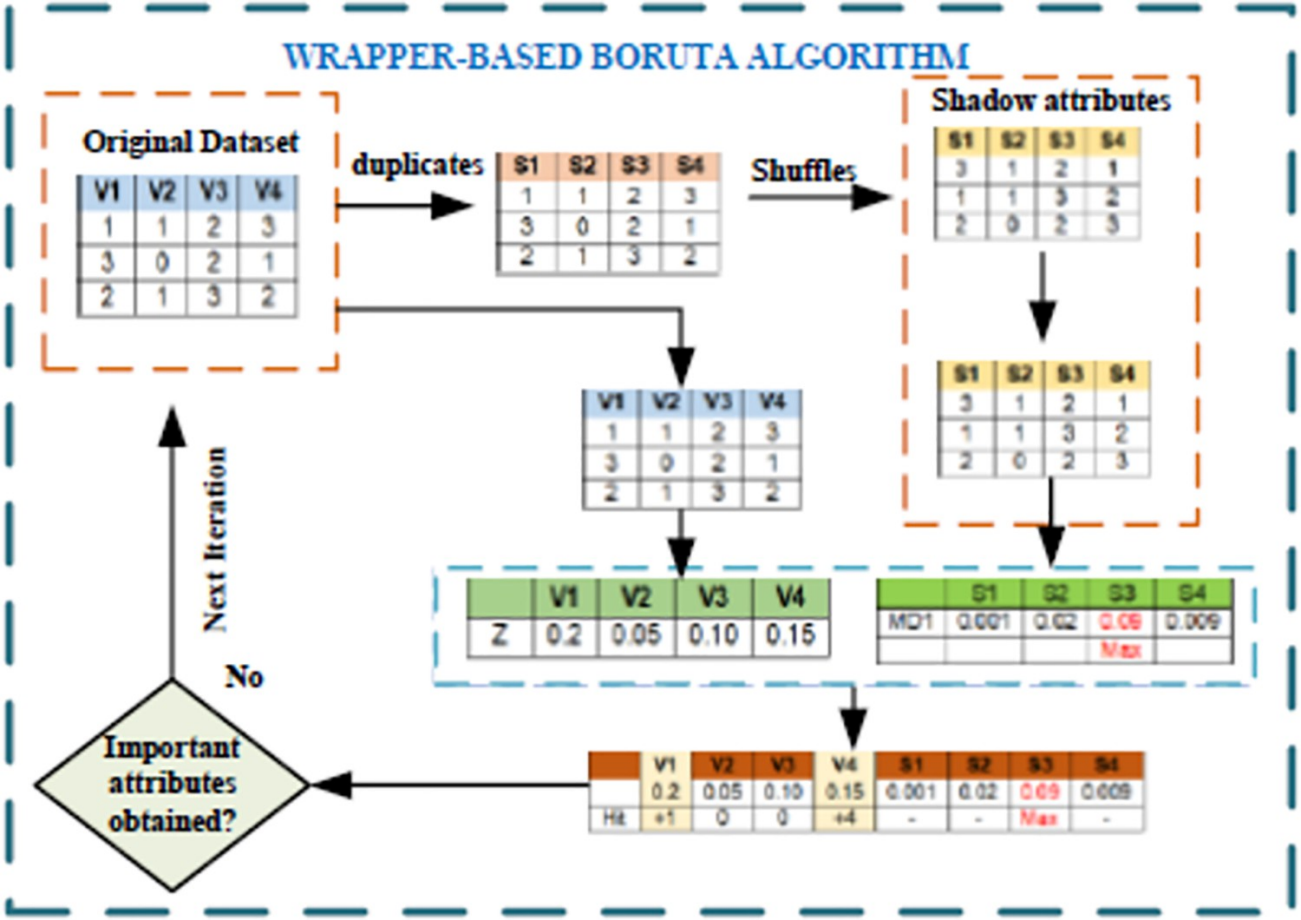
Wrapper-based Boruta Algorithm (BA).

One type of feature selection algorithm is the Boruta Algorithm (BA). This article uses the terms "features" and "attributes" interchangeably. The Random Forest (RF) algorithm [36] is wrapped in the BA algorithm. This algorithm is crucial when using a data set with multiple attributes to create a model. Unlike other popular feature selection algorithms, BA can choose all appropriate attributes from the attribute set, not just the ones that aren’t redundant. As a result, the attribute selection algorithm in this research was a wrapper-based BA. Finally, the attributes chosen are sent to various classifiers for further analysis. The process is depicted in Fig 2 and follows a step-by-step breakdown of how BA works.

- To begin, the algorithm randomizes the input dataset (***V***_1_,***V***_2_,***V***_3_,…,***V***_***n***_) by generating jumbled duplicates of all the attributes (***S***_1_,***S***_2_,***S***_3_,…,***S***_***n***_). These shuffled copies are called shadow attributes.
- The expanded dataset is then subjected to an attribute significance measure, and RF classifier is trained. Specifically, Mean Decrease Accuracy (MDI) is employed to quantify the significance of each attribute. The MDI quantifies the amount of accuracy that a classifier may lose by omitting each attribute. Therefore, the higher the MDI value for an attribute, the more important it is.
- It determines whether the original attribute is more important than the best of its shadow attribute at each iteration, that is, whether the attribute has a higher Z-score than the shadow attribute’s maximum Z-score and it removes attributes that are deemed highly unimportant. The Z-score is calculated by dividing the attribute’s classification accuracy loss by its standard deviation.
- The algorithm ends after several iterations when all attributes have been confirmed to be significant, tentative, or rejected, or when the specified RF classifier iterations have been reached.

### 2.5 Extreme gradient boosting (XGBoost)

Chen et al. [37] proposed the extreme gradient boosting (XGBoost) technique. The decision rules of XGBoost are very similar to those of a decision tree. It can be used for regression as well as classification. Although the gradient boosting machine (GBM) has recently been used in the field of traffic and transportation [38–41], XGBoost is a more efficient and scalable version of it. The main enhancements in XGBoost are the speed with which trees are created and the development of a novel distributed method for tree searches. The goal function’s value is optimized at the core of XGBoost. Given a dataset ***D*** = {(***x***_***i***_,***y***_***i***_)}, where ***x***_***i***_ denotes various dataset attributes and ***y***_***i***_ is the corresponding binary injury severity class (fatal and non-fatal). Assume that the XGBoost classifier is made up of *N*-decision trees, and that the optimization objective function is Eq 1.


$${\hat{y}}_{i}=\sum_{n=1}^{N}f_{n}(x_{i}),f_{n}\in R$$ (1)

Where, ***R*** is the regression tree space and each ***f***_***n***_ denotes independent tree with leaf scores. The Eq 2 provides the loss function.


$$L(f_{t})=\sum l_{f}({\hat{y}}_{i},y_{i})+\sum \chi (f_{t})$$ (2)

The differentiable loss function ***l***_***f***_, which measures the difference between the expected ${\hat{y}}_{i}$ and real outputs ***y***_***i***_, is the leading term in Eq 2. The regularization term ***χ*** is the second component, and it penalizes the models’ complexity to prevent over-fitting. Eqs 3 and 4 can be represented as expressions for ${\hat{y}}_{i}$ and ***χ***(***f***_***t***_), respectively.


$${\hat{y}}_{i}^{\left(t\right)}={\hat{y}}_{i}^{\left(t-1\right)}+f_{t}(x_{i})$$ (3)

$$\chi (f)=\gamma \psi +\frac{1}{2}{\left\|\tau \right\|}^{2}$$ (4)

Here, ***ψ*** is the number of leaf nodes, ***τ*** is each leaf score. Thus, we can derive Eq 5 as;

$$L(f_{t})\approx \sum_{j=1}^{T}\left[(\sum_{i\in I_{j}}g_{i})\tau_{j}+\frac{1}{2}(\sum_{i\in I_{j}}h_{i}+\lambda )\tau_{j}^{2}\right]+\gamma \psi$$ (5)

Here, ***g***_***i***_ and ***h***_***i***_ are 1^st^ and 2^nd^ order gradient statistics of the loss function. The ***γ*** and ***λ*** are constant parameters and they control the degree of regularization as well as prevent over-fitting.

The loss function’s 1^st^ and 2^nd^ order gradient statistics are ***g***_***i***_ and ***h***_***i***_. The ***γ*** and ***λ*** parameters are constants that govern the degree of regularization and prevent over-fitting.

### 2.6 K-Nearest Neighbor (K-NN)

The K-NN method, also known as neighbor-based classification (NBC), is a machine learning strategy based on supervised learning that is frequently used in traffic and transportation [42–44]. The K-NN classifies an observation in a prediction job by comparing it to the k observations that are closest to it. The nearest neighbor decision rule is used to assign a new sample point to a classification depending on which of a set of previously categorized points is closest to the new sample point. To put it another way, the vast majority of the k closest observations to the observation of interest should be included in the class of the observation of interest. Two decisions are necessary in the K-NN method: the value of k and the distance function, both of which are illustrated by Eq 6. This amount is usually established by experimenting with several values and determining which one delivers the best forecast accuracy as a consequence of the experimentation. The Euclidean distance, which may be conceived of as the distance between two points in two dimensions, is the basis for the K-NN distance function.


$$D_{e}=\sqrt{\left(x_{2}-x_{1})+(y_{2}-y_{1}\right)}$$ (6)

### 2.7 Binary Logistic Regression (BLR)

The association between a binary output parameter and one or more explanatory variables is modelled in binary logistic regression models. The explanatory variables are used in the logistic regression model to predict the likelihood that the response variable will take on a specific value. In binary logistic regression models, the response variable takes one of two binary values (0 or 1). The linear logistic regression model for a binary response variable y has the form shown in Eq 7.


$$\mathrm{logit}(P)=\ln(\frac{P}{1-P})=\beta_{0}+\beta_{1}k_{1},\ldots ,+\beta_{i}k_{i}+\varepsilon_{i}$$ (7)

The Eq 7 can be rewritten in terms of probability as Eq 8.


$$P=\frac{exp(\beta_{0}+\beta_{1}k_{1},\ldots ,+\beta_{i}k_{i})}{\left(1+\beta_{0}+\beta_{1}k_{1},\ldots ,+\beta_{i}k_{i}\right)}$$ (8)

Where,

***P***: the probability of fatal injuries

1−***P***: probability of non-fatal injuries

***k***_***i***_: ***i***^***th***^ attribute of model

***β***_***i***_: ***i***^***th***^ coefficient of model

***ε***: random error term

Fatal injuries on national highways are measured by the Odd Ratio (OR), which is defined as the probability of fatal injuries occurring on N-5 divided by the probability of non-fatal injuries occurring on national highways. The odds ratio (OR) is equal to exp(***β***_***i***_), which means that if the value of any component (***k***_***i***_) is increased by one unit while the values of all other components remain constant, the odds increase by an amount equal to the exp(***β***_***i***_). This illustrates how the relative quantity of the outcome either decreases (OR less than 1) or increases (OR greater than 1) depending on the condition.

### 2.8 Naïve Bayes (NB)

The Naive Bayes (NB) algorithm is one of the probabilistic classification techniques based on Bayes’ theorem, which assumes that the features are highly independent of one another. It is one of the most widely used algorithms. According to Jeong et al., [45], if the attribute vector (***V***_1_,***V***_2_,***V***_3_,…,***V***_***n***_) is given, the conditional probability of injury severity (fatal and non-fatal) can be expressed as Eq 9.


$$\begin{array}{l} P(C=c_{k}|x=(V_{1},V_{2},\ldots ,V_{n}))=\frac{P(C=c_{k}\cap^{x}=(V_{1},V_{2},\ldots ,V_{n}))}{P(X=(V_{1},V_{2},\ldots ,V_{n}))}\\ \;=\frac{P(X=(V_{1},V_{2},\ldots ,V_{n})|(C=c_{k})|P(C=c_{k}))}{P(X=(V_{1},V_{2},\ldots ,V_{n}))} \end{array}$$ (9)

It is also among the fastest classifiers available for large-scale data prediction and classification, and it is capable of handling both categorical and continuous data [46]. As a result, it has been demonstrated that NB is a simple and effective classification machine learning classifier for classification tasks.

### 2.9 Evaluation matrices

We compare the performance of different machine learning classifiers using the following evaluation criteria: Accuracy (ACC), F1-Measure, Receiver Operating Characteristic (ROC) Curve and AUC [47].

#### 2.9.1. Accuracy (ACC)

The "error rate" refers to the percentage of misclassified samples in comparison to total samples. If there are ’***q***’ misclassified samples among the total samples ’***p***’, the error rate is. ***Err*** = ***q***/***p***. Correspondingly, Eq 10 corresponds to the expression for computing accuracy (ACC).


$$ACC=\frac{TP+TN}{TP+TN+FP+FN}$$ (10)

#### 2.9.2. F1-measure

**Fig 3:**
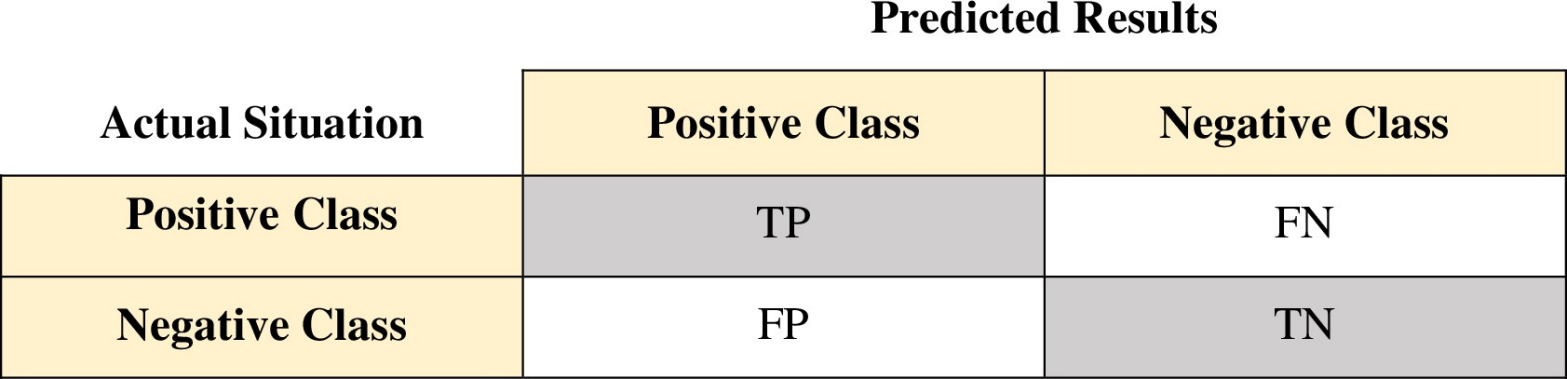
Confusion matrix.

ACC, despite its widespread use, does not meet all of the criteria. In the binary classification problem, samples are classified as true positive (TP), true negative (TN), false positive (FP), or false negative (FN) based on the combination of their actual and classifier projected classes. Fig 3 shows the confusion matrix.

Specificity and Sensitivity (also known as Recall) are two metrics described below. As defined in Eq 11, Specificity refers to the proportion of accurately predicted negative samples among all predicted negative class samples. Sensitivity is defined as the proportion of accurately predicted positive samples among all real positive class samples, as shown in Eq 12. The F1-measure is calculated as Eq 13.


$$\mathrm{Specificity}=\frac{\mathrm{TP}}{\mathrm{TP}+\mathrm{FN}}$$ (11)

$$\mathrm{Sensitivity}=\frac{\mathrm{TP}}{\mathrm{TP}+\mathrm{FP}}$$ (12)

$$\mathrm{F1}‐\mathrm{Measure}=\frac{2\mathrm{TP}}{2\mathrm{TP}+\mathrm{FP}+\mathrm{FN}}$$ (13)

#### 2.9.3. Receiver Operating Characteristic (ROC) curve and Area under the Curve (AUC)

The Receiver Operating Characteristic (ROC) curve is used to evaluate a classifier’s performance by plotting Sensitivity against Specificity. For binary classification problems, the AUC is used. It determines the two-dimensional area beneath the entire receiver operating characteristic curve.

## 3. Results and discussion

The injury severity in single-vehicle and multiple-vehicle accidents on N-5 was investigated using accident data from the National Highway and Motorway Police. To begin with, the missing values in the dataset were handled using a K-NN approach. In our datasets, a variety of factors influenced injury severity. The computational load on the classifier may have increased if all of them were included in the training model. The accuracy of the outcomes may be affected by correlations between attributes. To reduce the computational burden and improve classification accuracy, features must be reduced in dimensionality. The Boruta Algorithm (BA) was used to select the most influential attributes from the N-5 accident dataset for this purpose. After that, the influential attributes were used to predict injury severity using various statistical and machine learning classifiers.

### 3.1. Wrapper-based BA for the attributes selection

**Fig 4:**
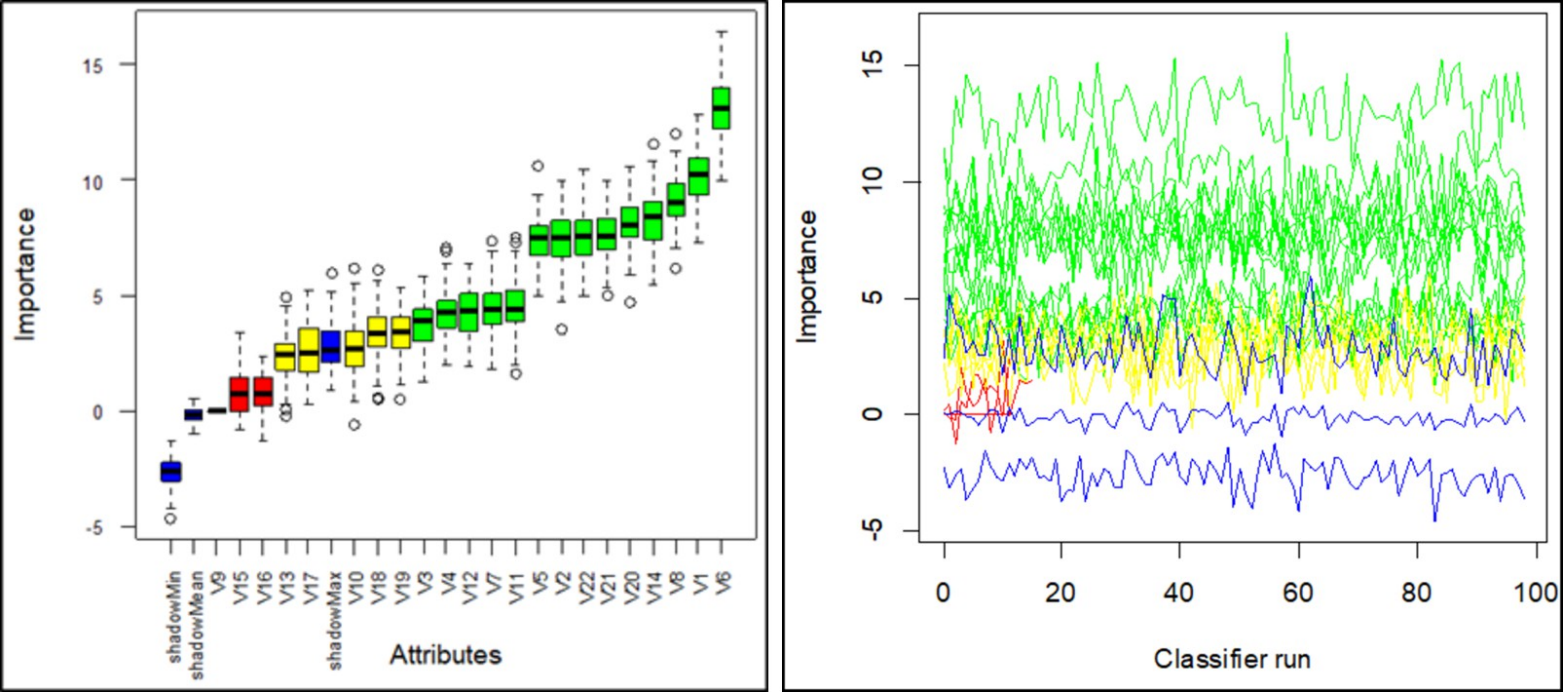
Boruta Algorithm outputs. **(a)** Box plots of attributes based on importance values. **(b)** Importance value of attributes in each classifier run.

We selected attributes from N-5 dataset that included both single vehicle and multiple-vehicle accidents. Using BA, it was determined that 14 of the 22 attributes were confirmed significant, four were tentative, and four were confirmed unimportant. The experimental analysis revealed that the vehicle type involved in the accidents (V6) was the most significant factor contributing to accidents on National Highway N-5 for both single and multiple vehicle accidents, with a mean importance value of 13.08 expressed as MDI according to BA. It is followed by the month of the year attribute (V1), which has a mean importance value of 10.13, the driver’s age (V8), which has a mean importance value of 9.13, and the alignment of the road segment (V14), which has a mean importance value of 8.23. The driver’s gender (V9) was confirmed unimportant, as was the presence of a median (V16) with a mean importance value of 0.75 and the presence of shoulder (V15) with a mean importance value of 0.82. A box plot of all attributes and their associated significance values is shown in Fig 4A. Blue boxplots represent the lowest, average, and highest Z-scores for each shadow attribute. The red, yellow, and green boxplots correspond to the Z-scores for rejected, tentative, and confirmed attributes, respectively. The tentative attributes can be classified as confirmed or rejected using the median Z-scores of the characteristics and the median Z-score of the best shadow attribute. Similarly, if the importance value falls between the blue and green lines, the attribute is more likely to be unimportant, whereas we have confirmed or significant attributes with much higher importance values than shadow attributes in the green area, shown by Fig 4A.

Table 2 summarizes several aspects of BA’s outputs. The Mean IMP column represents the mean of IMP, the Min-IMP column represents the minimum of IMP, the Median-IMP column represents the median of IMP, the Max IMP column represents the maximum of IMP, and the normHits column represents the number of hits normalized to the number of importance source runs, where IMP is the importance measure computed over multiple iterations of BA. For example, the value of normHits for V3 is 0.76, indicating that this attribute was found to be more important than shadow attributes 73% of the time and thus classified as a confirmed important attribute. The normHits of V1 is 1.00, which indicates that this attribute was found to be more important than shadow attributes 100% of the time and is thus considered a confirmed important attribute. However, the V15 normHits value is 0.01, indicating that this attribute is unimportant nearly 99% of the time. As a result, this attribute is considered confirmed unimportant and should be removed from further analysis.

**Table 2:** Attributes importance statistics by Boruta Algorithm (BA).

Attribute	Mean-IMP	Median-IMP	Min-IMP	Max-IMP	normHit	Decision about confirmation of Importance
**V1**	**10.13**	**10.23**	**7.27**	**12.84**	**1.00**	**Confirmed**
**V2**	**7.37**	**7.50**	**3.53**	**9.91**	**1.00**	**Confirmed**
**V3**	**3.74**	**3.93**	**1.23**	**5.78**	**0.76**	**Confirmed**
**V4**	**4.23**	**4.28**	**2.00**	**7.08**	**0.86**	**Confirmed**
**V5**	**7.40**	**7.50**	**4.97**	**10.59**	**1.00**	**Confirmed**
**V6**	**13.08**	**13.10**	**9.93**	**16.44**	**1.00**	**Confirmed**
**V7**	**4.42**	**4.41**	**1.85**	**7.39**	**0.89**	**Confirmed**
**V8**	**9.13**	**9.08**	**6.15**	**12.03**	**1.00**	**Confirmed**
V9	0.00	0.00	0.00	0.00	0.00	Rejected
V10	2.69	2.70	-0.57	6.15	0.45	Tentative
**V11**	**4.53**	**4.43**	**1.62**	**7.54**	**0.86**	**Confirmed**
**V12**	**4.34**	**4.31**	**1.89**	**6.43**	**0.86**	**Confirmed**
V13	2.38	2.39	-0.22	4.93	0.38	Tentative
**V14**	**8.23**	**8.40**	**5.46**	**11.55**	**1.00**	**Confirmed**
V15	0.82	0.70	-0.84	3.34	0.01	Rejected
V16	0.75	0.75	-1.29	2.35	0.00	Rejected
V17	2.61	2.50	0.26	5.25	0.42	Tentative
V18	3.32	3.40	0.49	6.14	0.65	Tentative
V19	3.42	3.43	0.53	5.36	0.64	Tentative
**V20**	**8.14**	**8.02**	**4.74**	**10.62**	**1.00**	**Confirmed**
**V21**	**7.63**	**7.59**	**5.03**	**9.90**	**1.00**	**Confirmed**
**V22**	**7.58**	**7.56**	**4.99**	**10.48**	**1.00**	**Confirmed**

**Fig 5:**
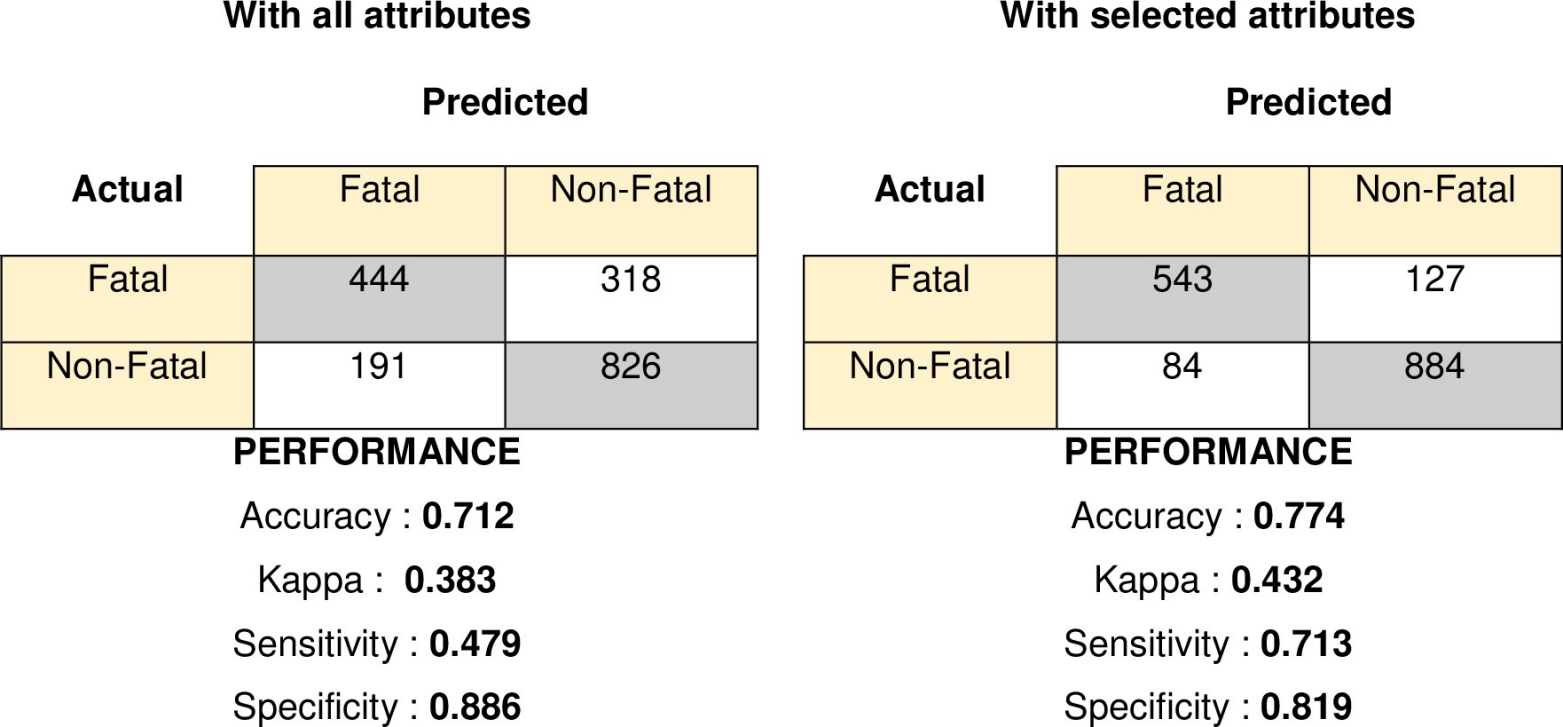
Confusion matrix of selected important attributes and all attributes.

Additionally, the Random forest (RF) classifier is used to predict the injury severity based on all of the attributes and on a subset of the confirmed important attributes. The accuracy was 71.20% when all attributes were used, but 77.41% when only selected attributes were used. Using selected attributes for injury severity prediction and classification improves accuracy by 6.20%. The confusion matrix obtained from the RF classifier with all and selected attributes is depicted in Fig 5. The confusion matrix is used to demonstrate the accuracy of injury severity prediction (fatal and non-fatal). The squares (1.1) and (2.2) in the confusion matrix for using all attributes and selected attributes indicate cases where the RF classifier correctly predicted, while the squares (1.2) and (2.1) indicate cases where the RF classifier incorrectly predicted. Considering both single and multiple-vehicle accidents and using all attributes, RF correctly classified 826 non-fatal and 444 fatal injuries out of 1144 non-fatal and 653 fatal injuries, respectively. In case of using only selected attributes, RF correctly classified 884 non-fatal injuries out of 1144 total non-fatal injuries and 543 fatal injuries out of total 653 fatal injuries.

### 3.2. Predictive performance comparison of different classifiers

The optimal attributes subset selected by wrapper-based BA was plugged into a set of four classifiers for further modeling. Using data from single and multiple vehicle accidents, we compared the XGBoost model to three other widely used classification approaches. A training set (80%) and a testing set (20%) were created from the accident datasets. Table 3 summarizes the overall performance of each machine-learning classifier as an average scoring metric.

**Table 3:** Comparison of prediction performance of different classifiers for single and multiple vehicles accidents.

Type of accident	Algorithm	Accuracy	Kappa	Sensitivity	Specificity	F1- Measure
**Single-vehicle accidents**	XGBoost	**0.821**	0.607	0.674	0.915	0.7762
	K-NN	0.781	0.527	0.689	0.813	0.745
	NB	0.576	0.026	0.061	0.961	0.114
	BLR	0.602	0.117	0.276	0.833	0.414
**Multiple-vehicle accidents**	XGBoost	**0.795**	0.569	0.641	0.910	0.752
	K-NN	0.682	0.354	0.643	0.711	0.675
	NB	0.576	0.026	0.061	0.961	0.114
	BLR	0.595	0.117	0.276	0.833	0.414

The following table compares four different machine learning classifiers for single- and multiple-vehicle accidents. According to these findings, the XGBoost classifier outperformed other approaches when only selected attributes were used to model. The accuracy of the XGBoost algorithm was 82.10%, the Cohen’s kappa was 0.607, the F1-Measure was 0.776, the Sensitivity was 0.674, and the Specificity was 0.915. It is followed by K-NN, which has a precision of 78.13%, a Cohen’s kappa of 0.527, an F1-Measure of 0.745, a sensitivity of 0.689, and a specificity of 0.813. The third is the BLR, which has a precision of 59.61%. The classifier with the worst performance is NB. It has a precision of 57.64% and a Cohen’s kappa of 0.607.

Additionally, we evaluated the performance of classifiers by examining all of the attributes associated with multiple vehicle accidents. It was observed that the XGBoost algorithm performed better for selected confirmed attributes, achieving an accuracy of 79.53%, Cohen’s kappa of 0.569, F1-Measure of 0.752, Sensitivity of 0.641, and Specificity of 0.910. Following that is K-NN, which has an accuracy of 68.21%, a Cohen’s kappa of 0.354, an F1-Measure of 0.675, a sensitivity of 0.643, and a specificity of 0.711. The third is the BLR, which has a precision of 59.61%. NB is the worst performing classifier. It has a precision of 57.64% and a Cohen’s kappa of 0.607. When all attributes were used in modelling, the accuracy of XGBoost, K-NN, NB, and BLR decreases by 5.80%, 5.00%, 2.30%, and 1.9%, respectively. It is clear that the XGBoost classifier, which was trained on single and multiple vehicle accident datasets, performed the best, while NB performed the worst. K-NN outperformed BLR and NB in terms of classification performance.

Along with classifier performance, we examined the predictive power of single-vehicle accident and multiple vehicle accident datasets on National Highway N-5. According to the classification results, single-vehicle accidents produced more accurate classifications than multiple vehicle crash datasets. On average, the classification accuracy, Cohen’s Kappa, F1-Measure, Sensitivity, and Specificity values for single-vehicle accidents using the XGBoost algorithm were 2.61%, 3.8%, 2.42%, 6.40%, and 3.05%, respectively, higher than the results from multiple vehicle accidents using the XGBoost algorithm.

**Fig 6:**
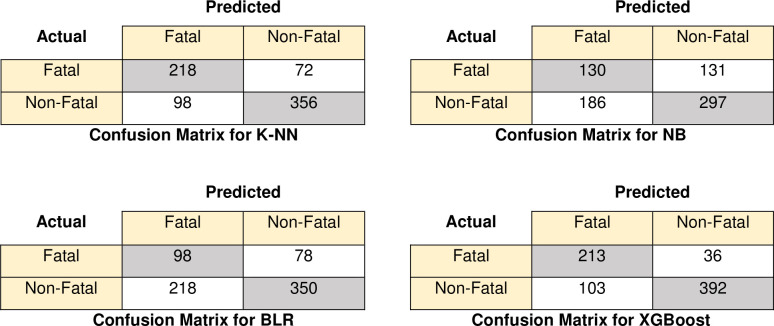
Confusion matrix for single-vehicle accidents using various classifiers.

**Fig 7:**
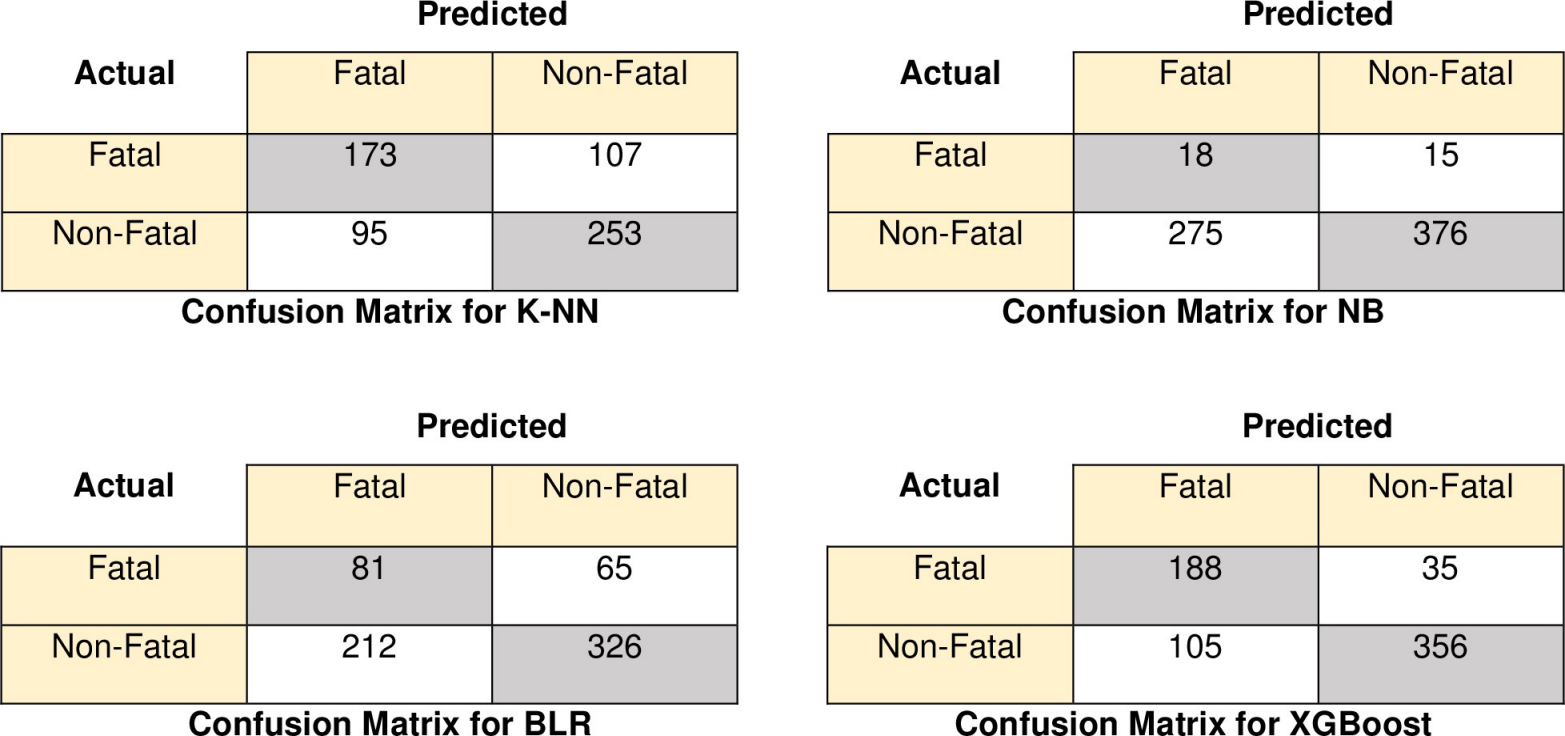
Confusion matrix for multiple-vehicle accidents using various classifiers.

Fig 6 and Fig 7 depict the confusion matrix for accidents involving single and multiple vehicles, respectively, for all classifiers using only confirmed attributes. In the case of single-vehicle accidents, the XGBoost classifier correctly predicted 392 injuries out of 428 non-fatal injuries, 213 injuries were accurately classified as fatal injuries. In the case of multiple vehicle collisions, the XGBoost classifier correctly predicted 356 injuries out of 392 non-fatal injuries and 188 injuries out 293 fatal injuries. K-NN was the second best classifier; thus, out of 428 non-fatal injuries, 356 non-fatal injuries were correctly classified, and 218 fatal injuries out of 316 fatal injuries were correctly classified for single-vehicle accident. Also, 253 non-fatal injuries, 173 fatal injuries were correctly predicted by K-NN in multiple vehicle accidents.

**Fig 8:**
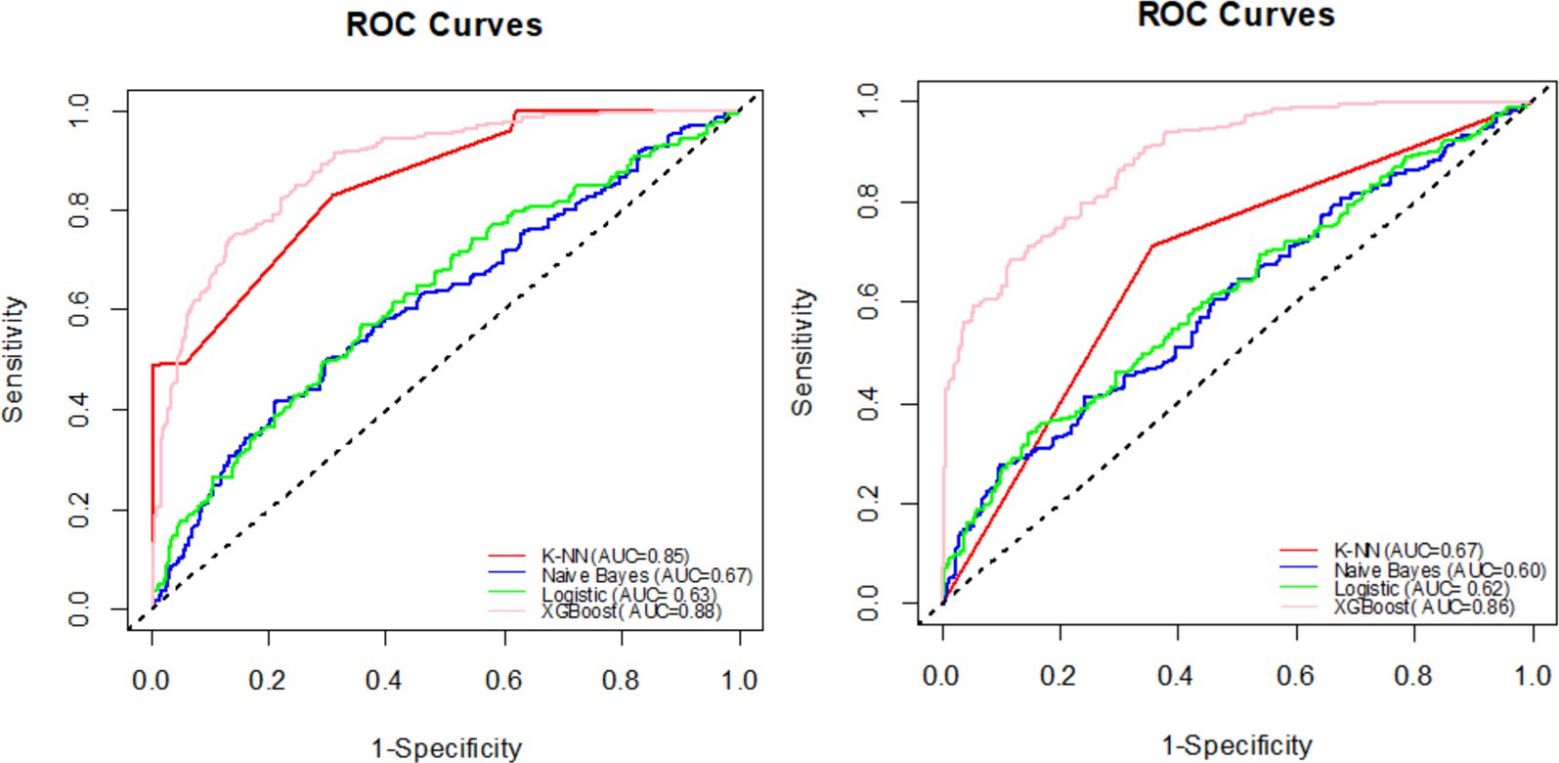
Comparison of AUC–ROC of different machine learning classifiers. **(a)** ROC curves for single-vehicles accidents. **(b)** ROC curves for multiple-vehicles accidents.

In order to compare our XGBoost approach to those of other machine learning classifiers, we plotted the receiver operating characteristic (ROC) curves of various classifiers. Fig 8 shows the AUC-ROC curves for four machine learning classifiers, illustrating the trade-off between sensitivity and specificity for different classifiers. For single-vehicle accidents, the XGBoost classifier performs better, with an AUC-ROC of 0.88. Likewise, the AUC-ROC curves for BLR (AUC-ROC = 0.63) and NB (AUC-ROC = 0.67) are closer to the 45-degree diagonal of the ROC space, indicating that these classifiers are less accurate. For multi-vehicle accidents, the XGBoost classifier performs better with an AUC-ROC of 0.86, followed by KNN with an AUC-ROC of 0.60, NB with an AUC-ROC of 0.62, and BLR with an AUC-ROC of 0.60.

## 4. Conclusions and recommendations

The aim of this paper is to use data from the National Highway and Motorway Police in Pakistan over a five-year period (2015–2019) to build hybrid feature-selection-based machine learning classifiers that can accurately predict the severity of road accidents. Single-vehicle accidents and multiple-vehicle accidents were first separated in the national highway N-5 accident dataset. The training and testing datasets were split 8:2. Because accuracy isn’t always the best way to judge machine learning classifiers, four more evaluation matrices were created, including Sensitivity, Specificity, Cohen’s Kappa, F1-Measure and to give a better idea of how well the classifiers did.

Using our proposed hybrid BA-based machine learning classification system, the BA first used random forest classifiers to find important attributes and confirmed important attribute using their MDIs. Out of 22 attributes, 14 were determined to be confirmed significant, four were determined to be tentative, and four were determined to be unimportant. The vehicle type was the most significant factor in crashes on national highway N-5, both single and multiple vehicle accidents, followed by the month of the year, the driver’s age, and the alignment of the road segment. The driver’s gender, the presence of a median, and the presence of shoulder were all determined to be insignificant. Unlike motorways in Pakistan for high speed traffic, N-5 is not an access-controlled highway but U-turns and at-grade intersections are provided at various locations with raised and depressed medians throughout. Only a few locations at urban areas have no medians. Similarly, at various locations shoulders are provided along the outer lanes. Therefore, due to their presence in most of the N-5 sections, both of these attributes have no impact on the injury severity in road accidents. Furthermore, most of the HTVs use N-5 for the freight transport from Karachi seaport to upcountry cities for long haul journey and LTVs for short haul inter-cities movement. Men drivers drive more miles in HTVs and likely to engage in risky driving than women and involve in accidents. Therefore, drivers’ gender also came out to be insignificant.

The comparison of various machine learning classifiers for single-vehicle accidents revealed that XGBoost achieved an accuracy of 82.10%, Cohen’s kappa 0.607, F1-Measure 0.776, Sensitivity 0.674, and Specificity 0.915, while the KNN achieved an accuracy of 78.13%, Cohen’s kappa 0.527, F1-Measure 0.745, Sensitivity 0.689, The classifier with the worst performance was NB, which had an accuracy of 57.64% and a Cohen’s kappa of 0.607.

XGBoost also performed better in multi-vehicle accidents, with an accuracy of 79.53%, Cohen’s kappa of 0.569, F1-Measure of 0.752, Sensitivity of 0.641, and Specificity of 0.910. The NB classifier once again demonstrated the lowest accuracy. KNN outperformed both NB and BLR. The hybrid Boruta Algorithm-based machine learning classification system may be beneficial for policymakers making safety decisions and for traffic safety researchers conducting large-scale analysis of traffic accidents in various locations. In comparison to more traditional models, it provides more information about accidents. The researchers may gain a better understanding of the various factors that contribute to accidents by employing this framework.

Finally, several recommendations are made for additional research. In future research, ensemble learning techniques such as model stacking may be used and the results compared to those obtained using other classifiers. Similarly, sensitivity analysis can be used to generate exhaustive interpretations for a variety of attributes. Additionally, motorway data could be gathered, and various attributes should be evaluated for their impact on Injury severity. The Synthetic Minority Oversampling Technique (SMOTE) can be used to deal with imbalanced datasets. This technique aids in overcoming the over-fitting problem. Additionally, it is recommended that the National Highway and Motorway Police develop a standard format for accident data collection that incorporates the aforementioned attributes.

## Supporting information

S1 Annexure (DOCX)

## References

[1] Lopez A.D.; Mathers C.D.; Ezzati M.; Jamison D.T., and Murray C.J., Global burden of disease and risk factors. 2006: The World Bank. 21250374

[2] Organization W.H., Global Status Report on Road Safety 2015. 2015: World Health Organization.

[3] Geedipally SR, Turner PA, Patil S. Analysis of motorcycle crashes in Texas with multinomial logit model. Transportation research record. 2011;2265(1):62–9.

[4] Chen Z, Fan WD. A multinomial logit model of pedestrian-vehicle crash severity in North Carolina. International journal of transportation science and technology. 2019 Mar 1;8(1):43–52.

[5] Vajari MA, Aghabayk K, Sadeghian M, Shiwakoti N. A multinomial logit model of motorcycle crash severity at Australian intersections. Journal of safety research. 2020 Jun 1;73:17–24. doi: 10.1016/j.jsr.2020.02.008 32563389

[6] Khattak A.J.; Pawlovich M.D.; Souleyrette R.R., and Hallmark S.L.J.J.o.T.E, Factors related to more severe older driver traffic crash injuries. 2002. 128(3): p. 243–249.

[7] Kockelman K.M. and Kweon Y.-J., Driver injury severity: an application of ordered probit models. Accident Analysis & Prevention, 2002. 34(3): p. 313–321. doi: 10.1016/s0001-4575(01)00028-8 11939360

[8] Abdel-Aty M., Analysis of driver injury severity levels at multiple locations using ordered probit models. Journal of safety research, 2003. 34(5): p. 597–603. doi: 10.1016/j.jsr.2003.05.009 14733994

[9] Rifaat S.M. and Chin H.C., Accident severity analysis using ordered probit model. Journal of advanced transportation, 2007. 41(1): p. 91–114.

[10] Xie Y.; Zhang Y., and Liang F., Crash injury severity analysis using Bayesian ordered probit models. Journal of Transportation Engineering, 2009. 135(1): p. 18–25.

[11] Garrido R.; Bastos A.; de Almeida A., and Elvas J.P., Prediction of road accident severity using the ordered probit model. Transportation Research Procedia, 2014. 3: p. 214–223.

[12] Rezapour M.; Moomen M., and Ksaibati K., Ordered logistic models of influencing factors on crash injury severity of single and multiple-vehicle downgrade crashes: A case study in Wyoming. Journal of safety research, 2019. 68: p. 107–118. doi: 10.1016/j.jsr.2018.12.006 30876502

[13] Wu Q, Chen F, Zhang G, Liu XC, Wang H, Bogus SM. Mixed logit model-based driver injury severity investigations in single-and multi-vehicle crashes on rural two-lane highways. Accident Analysis & Prevention. 2014 Nov 1;72:105–15. doi: 10.1016/j.aap.2014.06.014 25016459

[14] Chen F, Chen S, Ma X. Analysis of hourly crash likelihood using unbalanced panel data mixed logit model and real-time driving environmental big data. Journal of safety research. 2018 Jun 1;65:153–9. doi: 10.1016/j.jsr.2018.02.010 29776524

[15] Liu P, Fan W. Modeling head-on crash severity on NCDOT freeways: a mixed logit model approach. Canadian Journal of Civil Engineering. 2019;46(6):322–8.

[16] Chen F, Song M, Ma X. Investigation on the injury severity of drivers in rear-end collisions between cars using a random parameters bivariate ordered probit model. International journal of environmental research and public health. 2019 Jan;16(14):2632. doi: 10.3390/ijerph16142632 31340600 PMC6678079

[17] Zhang J.; Li Z.; Pu Z., and Xu C., Comparing prediction performance for crash injury severity among various machine learning and statistical methods. IEEE Access, 2018. 6: p. 60079–60087.

[18] Fiorentini N. and Losa M., Handling imbalanced data in road crash severity prediction by machine learning algorithms. Infrastructures, 2020. 5(7): p. 61.

[19] Wahab L. and Jiang H., Severity prediction of motorcycle crashes with machine learning methods. International journal of crashworthiness, 2020. 25(5): p. 485–492.

[20] Rahim M.A. and Hassan H.M., A deep learning based traffic crash severity prediction framework. Accident Analysis & Prevention, 2021. 154: p. 106090. doi: 10.1016/j.aap.2021.106090 33740462

[21] Lin C.; Wu D.; Liu H.; Xia X., and Bhattarai N., Factor identification and prediction for teen driver crash severity using machine learning: a case study. Applied Sciences, 2020. 10(5): p. 1675.

[22] Ahmadi A.; Jahangiri A.; Berardi V., and Machiani S.G., Crash severity analysis of rear-end crashes in California using statistical and machine learning classification methods. Journal of Transportation Safety & Security, 2020. 12(4): p. 522–546. doi: 10.4271/2016-01-1439 27648455 PMC5026383

[23] Galar M.; Fernandez A.; Barrenechea E.; Bustince H., and Herrera F., A review on ensembles for the class imbalance problem: bagging-, boosting-, and hybrid-based approaches. IEEE Transactions on Systems, Man, and Cybernetics, Part C (Applications and Reviews), 2011. 42(4): p. 463–484.

[24] Wang C.; Hu L.; Guo M.; Liu X., and Zou Q., imDC: an ensemble learning method for imbalanced classification with miRNA data. Genetics and Molecular Research, 2015. 14(1): p. 123–133. doi: 10.4238/2015.January.15.15 25729943

[25] Ji A. and Levinson D., Injury severity prediction from two-vehicle crash mechanisms with machine learning and ensemble models. IEEE Open Journal of Intelligent Transportation Systems, 2020. 1: p. 217–226.

[26] Jiang, L.; Y. Xie, and T. Ren. Modelling highly unbalanced crash injury severity data by ensemble methods and global sensitivity analysis. in Proceedings of the Transportation Research Board 98th Annual Meeting, Washington, DC, USA. 2019.

[27] Jalali-Heravi M. and Kyani A., Use of computer-assisted methods for the modeling of the retention time of a variety of volatile organic compounds: a PCA-MLR-ANN approach. Journal of chemical information and computer sciences, 2004. 44(4): p. 1328–1335. doi: 10.1021/ci0342270 15272841

[28] Abdul-Wahab S.A.; Bakheit C.S., and Al-Alawi S.M., Principal component and multiple regression analysis in modelling of ground-level ozone and factors affecting its concentrations. Environmental Modelling & Software, 2005. 20(10): p. 1263–1271.

[29] He F. and Ma C., Modeling greenhouse air humidity by means of artificial neural network and principal component analysis. Computers and Electronics in Agriculture, 2010. 71: p. S19–S23.

[30] Guillén-Casla V.; Rosales-Conrado N.; León-González M.E.; Pérez-Arribas L.V., and Polo-Díez L.M., Principal component analysis (PCA) and multiple linear regression (MLR) statistical tools to evaluate the effect of E-beam irradiation on ready-to-eat food. Journal of Food Composition and Analysis, 2011. 24(3): p. 456–464.

[31] Nasir M.F.M.; Samsudin M.S.; Mohamad I.; Awaluddin M.R.A.; Mansor M.A.; Juahir H., et al. River water quality modeling using combined principle component analysis (PCA) and multiple linear regressions (MLR): a case study at Klang River, Malaysia. World Applied Sciences Journal, 2011. 14: p. 73–82.

[32] Omid M.; Mahmoudi A., and Omid M.H., Development of pistachio sorting system using principal component analysis (PCA) assisted artificial neural network (ANN) of impact acoustics. Expert Systems with Applications, 2010. 37(10): p. 7205–7212.

[33] Chen, Z.-j.; L. Cheng; H.-n. Deng, and J.-k. Zhang, Analyzing Residential Travel Mode Choice Based on Principal Component Analysis, in ICCTP 2010: Integrated Transportation Systems: Green, Intelligent, Reliable. 2010. p. 2739–2746.

[34] Bham G.H.; Manepalli U.R., and Samaranayke V., A composite rank measure based on principal component analysis for hotspot identification on highways. Journal of Transportation Safety & Security, 2019. 11(3): p. 225–242. doi: 10.4271/2016-01-1439 27648455 PMC5026383

[35] Kassu A. and Hasan M., Identifying the principal factors influencing traffic safety on interstate highways. SN Applied Sciences, 2019. 1(12): p. 1–10.

[36] Ahmed AM, Deo RC, Feng Q, Ghahramani A, Raj N, Yin Z, et al. Deep learning hybrid model with Boruta-Random forest optimiser algorithm for streamflow forecasting with climate mode indices, rainfall, and periodicity. Journal of Hydrology. 2021 Aug 1;599:126350.

[37] Chen T, Guestrin C. Xgboost: A scalable tree boosting system. In Proceedings of the 22nd acm sigkdd international conference on knowledge discovery and data mining 2016 Aug 13 (pp. 785–794).

[38] Kalvapalli SP, Chelliah M. Analysis and Prediction of City-Scale Transportation System Using XGBOOST Technique. InRecent Developments in Machine Learning and Data Analytics 2019 (pp. 341–348). Springer, Singapore.

[39] Chen H, Chen H, Liu Z, Sun X, Zhou R. Analysis of factors affecting the severity of automated vehicle crashes using XGBoost model combining POI data. Journal of advanced transportation. 2020 Nov 19;2020.

[40] Yang C, Chen M, Yuan Q. The application of XGBoost and SHAP to examining the factors in freight truck-related crashes: An exploratory analysis. Accident Analysis & Prevention. 2021 Aug 1;158:106153. doi: 10.1016/j.aap.2021.106153 34034073

[41] Shi R, Xu X, Li J, Li Y. Prediction and analysis of train arrival delay based on XGBoost and Bayesian optimization. Applied Soft Computing. 2021 May 24:107538.

[42] Li Z, Han JL, Zhao XH, Zhu MH, Dong WH. Comparison of drunk driving recognizing methods based on KNN and SVM. Journal of transportation systems engineering and information technology. 2015;15(5):246–51.

[43] Li Z, Zhang Q, Zhao X. Performance analysis of K-nearest neighbor, support vector machine, and artificial neural network classifiers for driver drowsiness detection with different road geometries. International Journal of Distributed Sensor Networks. 2017 Sep;13(9):1550147717733391.

[44] Princess PJ, Silas S, Rajsingh EB. Classification of Road Accidents Using SVM and KNN. InAdvances in Artificial Intelligence and Data Engineering 2021 (pp. 27–41). Springer, Singapore.

[45] Jeong H, Jang Y, Bowman PJ, Masoud N. Classification of motor vehicle crash injury severity: A hybrid approach for imbalanced data. Accident Analysis & Prevention. 2018 Nov 1;120:250–61. doi: 10.1016/j.aap.2018.08.025 30173007

[46] Bhowmik TK. Naive bayes vs logistic regression: theory, implementation and experimental validation. Inteligencia Artificial. Revista Iberoamericana de Inteligencia Artificial. 2015;18(56):14–30.

[47] AlMamlook RE, Kwayu KM, Alkasisbeh MR, Frefer AA. Comparison of machine learning algorithms for predicting traffic accident severity. In2019 IEEE Jordan International Joint Conference on Electrical Engineering and Information Technology (JEEIT) 2019 Apr 9 (pp. 272–276). IEEE.

