# Supplementary material for: Hybrid feature selection-based machine learning Classification system for the prediction of injury severity in single and multiple-vehicle accidents
Source: PLoS One. 2022 Feb 2;17(2):e0262941. doi: 10.1371/journal.pone.0262941 (PMC8809572; doi:10.1371/journal.pone.0262941)
Supplement: S1 Annexure — (DOCX) [file pone.0262941.s001.docx]

**S1 Annexure**

| **ATTRIBUTES** | **ATTRIBUTES DESCRIPTION** | **FREQUENCY** | **MARGINAL PERCENTAGE (%)** |
| --- | --- | --- | --- |
| **Injury Severity (Y)** | 0= Fatal  1= non-Fatal | 761  1023 | 42.66%  57.34% |
| **Month of the Year (V1)** | 1= January  2= February  3= March  4= April  5=May  6= June  7= July  8= August  9= September  10= October  11= November  12= December | 118  143  173  208  132  160  162  176  169  102  118  123 | 6.61%  8.02%  9.70%  11.66%  7.40%  8.97%  9.08%  9.87%  9.47%  5.72%  6.61%  6.89% |
| **Day of the Week (V2)** | 1= Monday  2= Tuesday  3= Wednesday  4= Thursday  5=Friday  6= Saturday  7= Sunday | 192  226  259  241  301  300  265 | 10.76%  12.67%  14.52%  13.51%  16.87%  16.82%  14.85% |
| **Type of Day (V3)** | 1= Weekday  2= Weekend | 1217  567 | 68.22%  31.78% |
| **Day/Night (V4)** | 0= Night  1= Day | 540  1244 | 30.27%  69.73% |
| **Time of Accident (V5)** | 1= 12:00:00 AM - 3:59:59 AM  2= 4:00:00 AM - 7:59:59 AM  3= 8:00:00 AM - 11:59:59 PM  4= 12:00:00 PM - 3:59:59 PM  5= 4:00:00 PM - 7:59:59 PM  6= 8:00:00 PM - 11:59:59 PM | 160  257  412  377  375  203 | 8.97%  14.41%  23.09%  21.13%  21.02%  11.38% |
| **Vehicle Type Involved in accidents (V6)** | 1=Bicycle  2=Motorcycle  3=Rickshaw  4=Car  5=Pickup  6=Minibus  7=Bus  8=Truck  9=Dumper  10=Trailer  11=Tractor | 0  121  15  262  116  164  156  501  79  329  41 | 0.00%  6.78%  0.84%  14.69%  6.50%  9.19%  8.74%  28.08%  4.43%  18.44%  2.30% |
| **Age of Vehicle (Yrs.) (V7)** | 1=0-10  2=11-20  3=21-30  4=31-40  5=41-54 | 571  651  273  166  123 | 32.01%  36.49%  15.30%  9.30%  6.89% |
| **Age of Drivers (Yrs.) (V8)** | 1=18-25  2=25-30  3=31-35  4=36-40  5=41-45  6=46-50  7=51-55  8=55-70 | 282  275  278  309  256  213  96  75 | 15.81%  15.41%  15.58%  17.32%  14.35%  11.94%  5.38%  4.20% |
| **Gender of Driver (V9)** | 0= Female  1= Male | 3  1781 | 0.17%  99.83% |
| **Driving License (V10)** | 0= No  1= Yes | 830  954 | 46.52%  53.48% |
| **Lighting Condition (V11)** | 1=Day Light  2=Night with Road Lights  3=Night without Road Lights | 1233  95  456 | 69.11%  5.33%  25.56% |
| **Weather Condition (V12)** | 1=Sunny  2=Cloudy  3=Rainy | 1603  64  117 | 89.85%  3.59%  6.56% |
| **Visibility Condition (V13)** | 1=Clear  2=Fog  3=Smog | 1720  55  9 | 96.41%  3.08%  0.50% |
| **Road Segment Alignment (V14)** | 1=Straight  2=Horizontal Curve  3=Vertical Curve  4=Horizontal and Vertical Curve | 1505  101  79  99 | 84.36%  5.66%  4.43%  5.55% |
| **Shoulder Presence (V15)** | 0= No  1= Yes | 47  1737 | 2.63%  97.37% |
| **Median Presence (V16)** | 0= No  1= Yes | 65  1719 | 3.64%  96.36% |
| **Surface Condition (V17)** | 1= Dry  2= Wet | 1650  134 | 92.49%  7.51% |
| **Surface Smoothness (V18)** | 1= Smooth  2= Rough  3=Potholes | 1681  45  58 | 94.23%  2.52%  3.25% |
| **Road Type (V19)** | 1= Urban  2= Rural | 943  841 | 52.86%  47.14% |
| **Collision Type (V20)** | 1=Head on collision  2=Rear End Collision  3=Side Collision  4=Roll Over  5=Skid  6=Hit Obstruction  7=Hit pedestrian  8=Hit Animal  9=Run off Roadway  10=Tire Burst  11=Fell of Bridge | 93  777  342  222  55  87  184  5  14  2  3 | 5.21%  43.55%  19.17%  12.44%  3.08%  4.88%  10.31%  0.28%  0.78%  0.11%  0.17% |
| **Work Zone (V21)** | 0= No  1= Yes | 997  787 | 55.89%  44.11% |
| **Apparent Cause (V22)** | 1=Bicycle rider Fault  2=Wrong Side Over taking  3=Pedestrian Fault  4=Road surface distress  5=Driver Fault  6=Dozing at wheel  7=Over Speeding  8=Motorcycle Rider Fault  9=Fog  10=Vehicle Fault  11=Mechanical Fault  12=Slippery Road  13=Tire Burst  14=Driver Sleeping  15=other | 10  26  130  27  1005  25  69  56  7  138  32  42  16  42  159 | 0.56%  1.46%  7.29%  1.51%  56.33%  1.40%  3.87%  3.14%  0.39%  7.74%  1.79%  2.35%  0.90%  2.35%  8.91% |
